# A hybrid living/organic electrochemical transistor based on the *Physarum polycephalum* cell endowed with both sensing and memristive properties

**DOI:** 10.1039/c4sc03425b

**Published:** 2015-02-20

**Authors:** G. Tarabella, P. D'Angelo, A. Cifarelli, A. Dimonte, A. Romeo, T. Berzina, V. Erokhin, S. Iannotta

**Affiliations:** a IMEM-CNR , Institute of Materials for Electronics and Magnetism – National Research Council , Parco Area delle Scienze 37/A – 43124 , Parma , Italy . Email: dangelo@imem.cnr.it ; Email: iannotta@imem.cnr.it

## Abstract

A hybrid bio-organic electrochemical transistor was developed by interfacing an organic semiconductor, poly(3,4-ethylenedioxythiophene) doped with poly(styrene sulfonate), with the *Physarum polycephalum* cell. The system shows unprecedented performances since it could be operated both as a transistor, in a three-terminal configuration, and as a memristive device in a two terminal configuration mode. This is quite a remarkable achievement since, in the transistor mode, it can be used as a very sensitive bio-sensor directly monitoring biochemical processes occurring in the cell, while, as a memristive device, it represents one of the very first examples of a bio-hybrid system demonstrating such a property. Our system combines memory and sensing in the same system, possibly interfacing unconventional computing. The system was studied by a full electrical characterization using a series of different gate electrodes, namely made of Ag, Au and Pt, which typically show different operation modes in organic electrochemical transistors. Our experiment demonstrates that a remarkable sensing capability could potentially be implemented. We envisage that this system could be classified as a Bio-Organic Sensing/Memristive Device (BOSMD), where the dual functionality allows merging of the sensing and memory properties, paving the way to new and unexplored opportunities in bioelectronics.

## Abstract 2

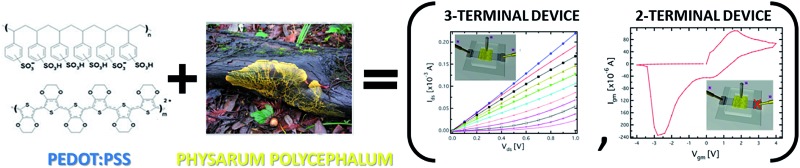
A hybrid bio-organic electrochemical transistor based on the *Physarum polycephalum* cell, showing a multifunctional operation (transistor and memristive-like response), has been demonstrated.

## Introduction

One of the main interests in bioelectronics, besides the relevant expected impacts in bio-medicine and prosthetics, is driven by the aim to emulate abilities that, essentially present in living beings, can scarcely be reproduced with artificial man-made devices. Even the simplest living organisms, for example, learn from and adapt themselves to stimuli from the surrounding environment. A challenging perspective is the integration of these adaptive/learning behaviours into artificial systems, possibly interfacing them with existing devices and technologies. Even though the great efforts of the scientific community in this direction have brought enormous progress in interfacing living beings with electronic devices, currently artificial models can barely mimic the basic properties of the simplest living organism in an oversimplified way.^1^ Remarkable state of the art work also aims at demonstrating the feasibility of biodevices and bio-inspired systems.^2,3^

In this framework, quite a relevant evolution has been determined by the concept of a memristor, introduced theoretically^4^ and hence realized experimentally.^5^ The basic concept underlying a memristor involves a device inherently endowed with memory, the resistance of which can switch from an insulating to a conductive state, depending on the sequence of electrical signals experienced. Hence, a memristor is particularly well suited for mimicking the learning behaviour of biosystems, opening novel perspectives in information processing.^6^

In bioelectronics, a strongly evolving novel strategy is based on organic electronics and in particular on organic electrochemical transistors (OECTs).^7–9^ OECTs are very promising as biocompatible sensing devices,^10–12^ as electrodes interfacing neurons^13^ and nervous systems, as well as active elements for bioelectronics,^14^ and for the recently proposed *iontronics* (ion-based signal handling and processing including bio-actuation).^15,16^ Organic bioelectronic devices, based on organic semiconductors, are increasingly attracting the scientific community since they operate with electrolytes in the liquid phase and at low bias voltages (<1 V), and are fully biocompatible.^7,8^

An OECT consists essentially of a semiconducting polymer channel in contact with an electrolyte, properly confined by a PDMS-well. The gate electrode is immersed into the electrolyte and the overlapping area between the organic polymer and the electrolyte defines the channel of the OECT, where the ionic interchanges can take place.^8^ At present, the most popular conducting polymer is poly (3,4-ethylenedioxythiophene) doped with poly(styrene sulfonate), PEDOT:PSS. The operating mechanism of the OECT is based on the reversible doping/de-doping of the channel: upon application of a drain–source voltage *V*_ds_, holes drift within the transistor channel, generating a drain–source current *I*_ds_ (the *on* state); when a positive voltage *V*_gs_ is applied, cations M^+^ from the electrolyte penetrate into the PEDOT:PSS channel and de-dope it according to a redox reaction (the *off* state).^17,18^ Even though the electrolyte is often a simple physiological solution, such as NaCl or phosphate buffered saline (PBS),^19^ OECTs based on PEDOT:PSS work efficiently even with more complex solutions, such as cell culture media,^19^ solid-gels^20^ and micellar electrolytes,^21^ hence becoming a suitable playground for addressing very relevant questions concerning cell functioning,^11,12^ signalling and stress, drug delivery systems and processes,^10^ neuronal and brain functions and working principles (including synaptic and post-synaptic processes).^13,22^

We report here on a novel hybrid bioelectronic organic electrochemical device based on a living being – the *Physarum polycephalum* cell (PPC), a multinuclear single-cell mass of protoplasm belonging to the family of myxomycetes, in the past defined as fungi, nowadays simply slime moulds. PPC lives in humid and dark environments. The studied form, in particular, is the plasmodium, PPC vegetative form; it looks like an amorphous yellow mass with networks of protoplasmic tubes branching towards nutrients. Its foraging behaviour can be seen as a computation: data are represented by spatial configurations of attractants and repellents, and results by the structure of protoplasmic networks.^23^ Therefore, PPC is widely studied for unconventional computing^24^ applications as it has demonstrated the capability of resolving optimization problems.

We demonstrate that this hybrid “living” device operates reproducibly both as a transistor and as a memristive device, and its peculiar features pave the way to novel strategies based on the integration of organic bioelectronics with memristive approaches. The choice of PPC was based on its unique, recognized properties of “intelligence”, “creativity” and “capacity of learning” that are being increasingly investigated.^25^ For example, during its life cycle, and especially when it seeks food, PPC is able to remember already trodden paths, in order not to retrace them. For this reason, PPC has recently been exploited as the main material in non-conventional computing, robot-*Physarum* and PPC-based network circuits.^26–28^ In addition, it is rather easy to keep the PPC alive: it requires room temperature, dark conditions and humidity. The PPC colony needs to be fed with oat flakes and periodically replanted to fresh substrates. Therefore, given a little care and constant attention it is possible to grow PPC in its yellow plasmodial stage.

## Materials and methods

### OECT fabrication

The OECT channel was made of PEDOT:PSS, a p-type semiconductive polymer widely used in bioelectronics because of its demonstrated properties of stability and biocompatibility.^29,30^ Before patterning, the solution was doped with diethylene glycol 20% (Sigma) with 0.05% by vol. of dodecyl benzene sulfonic acid (DBSA) surfactant (Sigma Aldrich), in order to enhance its electrical conductivity and film-forming properties, respectively.^31,32^ The OECT channel, with a final width of 2 mm, was patterned on a square glass slide of 2 × 2 cm and the PEDOT:PSS was spun onto the substrate using a first ramp of 6 s (at 450 rpm) followed by a 30 s plateau at 1500 rpm. The final film thickness was *d* ∼ 100 nm, as measured using a profilometer. Devices were finally baked on a hotplate at 120 °C for 120 min. A PDMS well of about 500 μL in volume was used to confine the mould onto the channel, defined by the overlapping area of the mould with the PEDOT:PSS stripe.

### Culture of *Physarum polycephalum*

Plasmodium of *Physarum polycephalum* was cultivated in a glass box, kept in a dark, humidifying atmosphere with a water bath, on wet towels and fed with oat flakes. Cultures were periodically replanted to a fresh substrate. Fresh PPC was placed into the PDMS well for each electrical measurement. Therefore, after setting up the OECT, a 150 μl blob of fresh PPC was picked from the growing box and manually inserted into the PDMS well. The operation was carried out with the help of a small spatula taking care that the mould made contact with the underlying PEDOT:PSS channel. Subsequently, the gate electrode was inserted into the PPC's body without touching the polymeric channel. All the measurements were performed under dark conditions in order to preserve the PPC. More details on the culture growth can be found in literature.^33^

### OECT electrical characterization

**Fig. 1:**
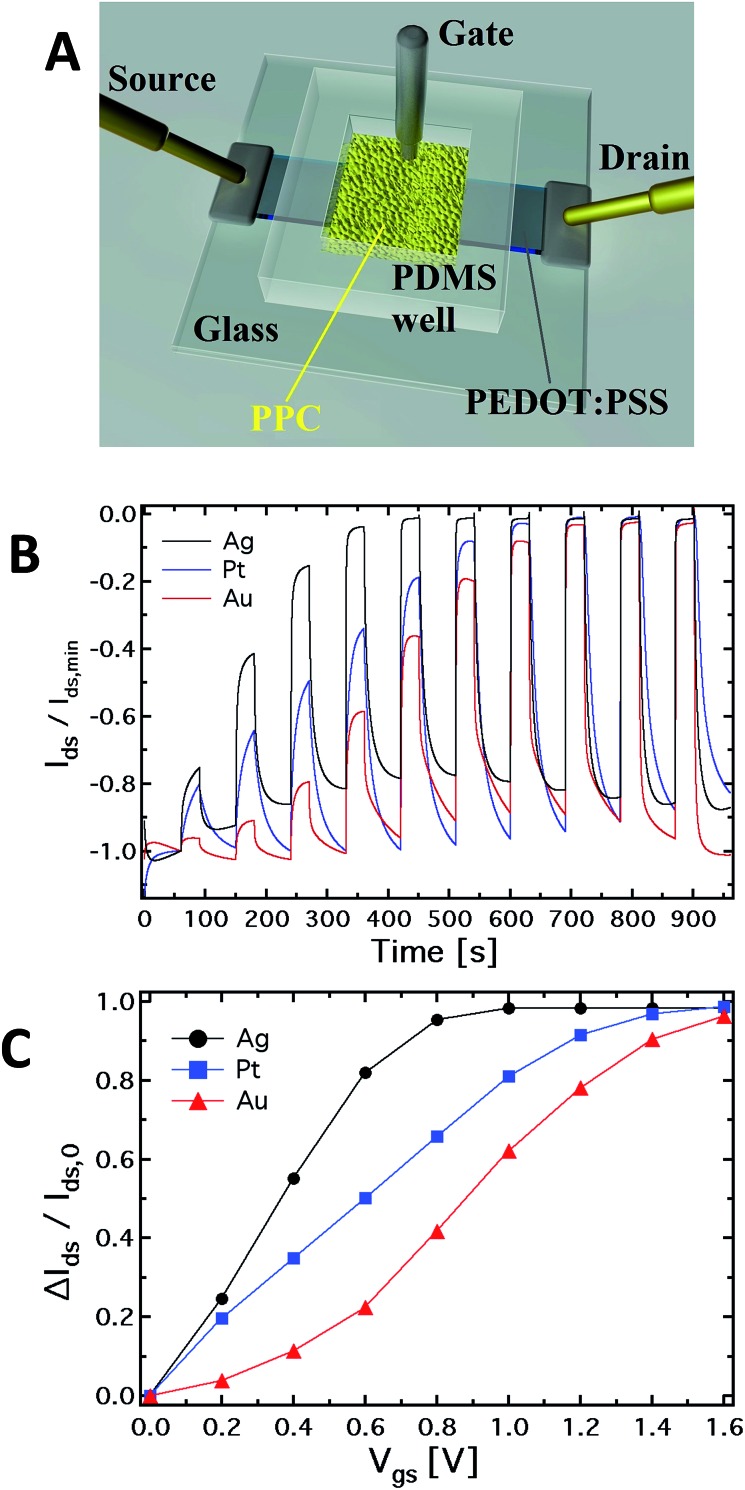
(A) Schematic diagram of the OECT based on PEDOT:PSS (the black stripe is the PEDOT:PSS film) where the gate electrode is immersed into the *Physarum polycephalum* cell (the yellow area in the figure). (B) Normalized kinetic curves (*I*_ds_/*I*_ds,min_ *vs.* time) and corresponding (C) current modulation (Δ*I*/*I*_0_): each step corresponds to an increment of 0.2 V of the gate voltage.

Electrical measurements were carried out using a 2 channel source/measure precision unit (Agilent B2902A), controlled by home-made LabView software. Before the experiments, the OECT channel was immersed in DI water for 1 hour in order to properly hydrate the PEDOT:PSS layer, while the gate electrodes were cleaned to remove any residue. Two types of measurements were carried out: in the first set, a 3-electrode device was used under a transistor-mode configuration (see Fig. 1A), recording the output and transfer characteristics. In the second set of measurements, the source electrode was interdicted, and a 2-terminal device was exploited to carry out *I*–*V* cyclic measurements, with the polymeric film used as the reference electrode and the metal wire as the working electrode. The PPC-OECT device was tested acquiring the typical output and transfer characteristics, as well as the kinetic curves.

Hereafter, we define the “kinetic curve” as the measurement of source–drain current (*I*_ds_) *vs.* time recorded under a constant drain voltage (*V*_ds_ = –0.4 V) and by varying the gate voltage *V*_gs_ according to a step-like scan mode, that is by applying voltage steps in the range 0–2 V with step heights increasing progressively by 0.2 V. Kinetic curves are generally acquired by operating OECTs in sensor mode in order to extract the modulation ratio Δ*I*/*I*_0_ = (*I* – *I*_0_)/*I*_0_, where *I*_0_ is the current value for *V*_gs_ = 0 V, and *I* is the current value for *V*_gs_ > 0 V. The modulation ratio is the typical parameter for quantifying the performance of OECTs used in sensor mode.^34,35^ In addition, the transfer curves show the channel current *I*_ds_ flowing between the source and drain electrodes as a function of the gate voltage (*V*_gs_) under a constant drain voltage *V*_ds_ = –0.4 V. On the other hand, output characteristics consist of the channel current *I*_ds_ recorded as a function of the drain voltage *V*_ds_ under a constant (variable) gate voltage *V*_gs_, resulting thus in a set of *I*_ds_ *vs. V*_ds_ curves parameterized by *V*_gs_. The gate current was simultaneously acquired during all the measurements. In order to obtain a steady-state curve, each point of the channel current was acquired with a delay of 30 s after the voltage application. Finally, a 2-terminal device was used to investigate the electrochemical response of the different electrodes inserted into the PP cell. This set of measurements was performed applying a bias between the PEDOT:PSS film and the mould, contacting the electrode inserted into it. The measurements were carried out *via* a series of voltage scans with steps of 0.2 V separated by a 10 s delay: the first range is between 0 and 2 V, followed by a scan between +2 to –2 V, and finally from –2 V to 0 V. Control experiments have shown that the interaction of the gate electrode with the mould does not affect the viability of the PPC because, after insertion, the membrane of the PPC rearranges itself in a new state of equilibrium.

## Results and discussion

Our extensive study of the electrochemical transistor (hereafter referred as PPC-OECT) has been carried out by systematically changing the gate electrode material, that is platinum (Pt), silver (Ag) and gold (Au). In fact it has been recognized that the nature of the gate electrode can affect the device response in quite a relevant way, due to the different electrochemical reactivity between the metallic electrode and electrochemically active species.^17^ On the basis of the results reported in ref. 17, we have tried to highlight the connection between the electrical response of the device and intracellular mechanisms driven by electrochemical processes in the body of the PPC.

We first made a full standard electrical characterization by performing electrical measurements in a transistor-mode configuration (output and transfer characteristics). This characterization demonstrated the OECT-like operation. Then, we performed cyclic current–voltage measurements (*I*–*V*), by using the device in a 2-terminal configuration, where the OECT organic semiconducting layer works as the reference electrode and the metal gate plays the role of the working electrode.

A major result of our work is that the PPC-OECT shows operation as a multifunctional device, consisting of both a transistor-like and a memristor response, and is fully satisfactory for proper implementation as a memristive element. The switching between the OECT mode and the memristor mode is realized by interdicting the drain electrode. Such a hybrid device, based on interfacing organic electronics with living systems, is ideally suitable for building artificial bio-inspired systems. At the same time, the hybrid living/organic interface is an ideal electrochemically-active microenvironment suitable for studying *in situ* and in *real-time* both intracellular bioprocesses (whether induced or not by interaction with the environment) as well as collective properties of living organisms, including self-replicating systems. We use a living organism as an active device element, so that our PPC-OECT represents a prototypal test architecture aimed at showing the possibility to ideally scale down OECTs towards microscopic structures and, at the same time, a tool suitable to study membrane effects, eventually related, for example, to specific pathologies. Moreover, the memristive device counterpart can be used in a multifunctional device view for the recording/storing of specific cellular activities.

### The PPC-OECT transistor performance

Fig. 1A shows a schematic of the hybrid PPC-OECT structure, where the yellow area represents the slime mould, while the black stripe between the source and drain electrodes represents the PEDOT:PSS film and the gate electrode is placed inside the PPC.

Fig. 1B shows the typical kinetic curves, *I*_ds_(*t*), measured by switching the gate voltage in the range 0–1.6 V with steps of 0.2 V. The device response upon application of gate voltage steps is defined by the modulation ratio Δ*I*/*I*_0_ = (*I* – *I*_0_)/*I*_0_. The comparison among the typical current modulations observed for the three different gate electrodes is reported in Fig. 1C. As already indicated above, generally, Δ*I*/*I*_0_ *vs. V*_gs_ curves are used for expressing the sensing capability of OECTs in the presence of different analytes in an aqueous suspension, and/or different concentrations for a given analyte.

In our case, the comparison in Fig. 1C is used in order to show in a clear way how the mould reacts electrochemically in the presence of different gate electrode materials. In this respect, it is known that an OECT can work in two operating regimes, namely Faradaic and non-Faradaic (capacitive).^17,36^ In the Faradaic mode, a redox reaction occurs at the gate surface, generating a current in the gate–source circuit that decreases the potential drop at the gate/electrolyte interface, therefore increasing the effective gate voltage (*V*effg) acting on the transistor channel.

Consequently, an increasing amount of ions is injected into the polymer film, de-doping it and hence inducing a significant decrease of the source–drain current, which, in turn, results in an increased current modulation. When the capacitive mode is dominant, an electrical double layer is formed at the gate/electrolyte interface. In this case, a significant potential drop arises at this interface, which reduces the *V*effg acting on the polymeric film underneath, therefore limiting its de-doping.

As previously observed, the electrode material determines the electrochemical regime under which the PPC-OECT operates. As expected,^17,37^ the Ag gate, being redox-active, generates the highest current modulation over the whole voltage range investigated. On the other hand, Pt and Au, both without any significant redox reactivity, show lower current modulations. For instance, Δ*I*/*I*_0_ at *V*_gs_ = 0.8 V is 0.41 for the Au-gate, 0.65 for the Pt-gate and 0.95 for the Ag-gate. In the PPC-OECT a non-Faradaic regime is hence expected when a Pt or an Au gate are used. Fig. 1C shows the progressive gate voltage shift towards lower gate voltages of the transfer curves depending on the material of the gate electrode (Au < Pt < Ag), indicating that the *V*effg increases progressively. In particular, focusing on the modulation value Δ*I*/*I*_0_ of 0.5, the voltage gate shift between the Ag and Au gates is ∼0.5 V, whereas that between Pt and Au is ∼0.2. This is the effect induced by the specific reactivity of the gate material with the saline environment inside the cell, confirming what we expected from the abovementioned electrochemical considerations. These findings already give a first strong indication that the PPC-OECT provides valuable and direct “*in situ*” information about the electrochemical state of the mould cell itself and hence, possibly, of its interaction with the environment.

**Fig. 2:**
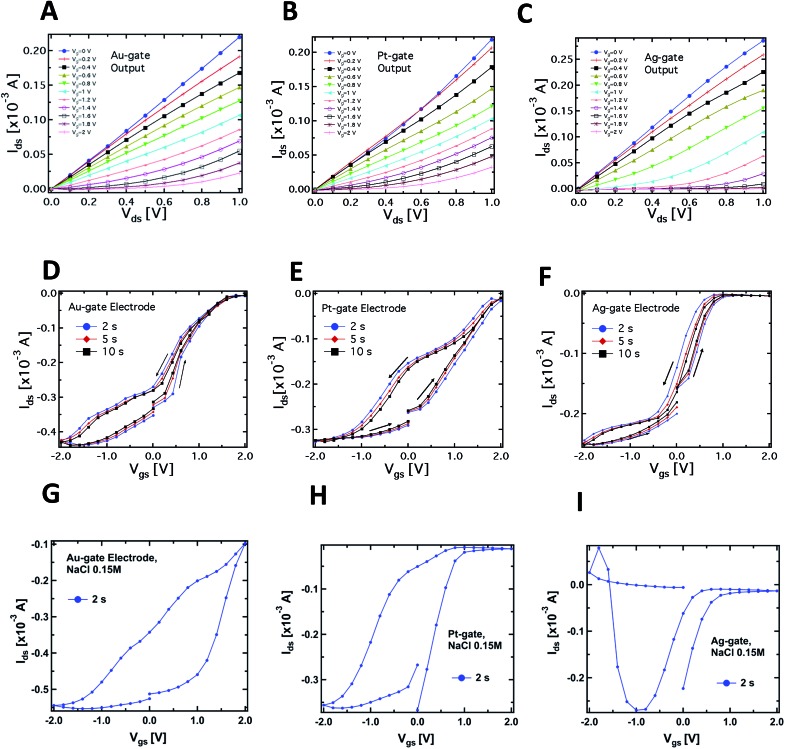
Typical output characteristics (*I*_ds_ *vs. V*_ds_ at different *V*_gs_) of the PPC-OECT device recorded by using Au (A), Pt (B) and Ag (C) wires as gate electrodes inserted into the PPC. Corresponding transfer characteristics (*I*_ds_ *vs. V*_gs_) for Au, Pt and Ag gate electrodes (*V*_ds_ = –0.4 V) are reported in panels D, E and F, respectively. The last three panels show the transfer characteristics acquired using a standard NaCl solution as electrolyte (0.15 M) and Au (G), Pt (H) and Ag (I) gate electrodes.

Fig. 2A–C compare the typical output characteristics (*I*_ds_ *vs. V*_ds_ at different *V*_gs_) for the three different gate electrodes investigated. In all cases, the output curves show that the devices work properly as transistors, operating in depletion mode in a very similar way to more standard electrolyte-gated transistors (even though the saturation regime occurring at voltages higher than 1 V was not investigated in order to avoid water electrolysis). We have observed an excellent biocompatibility of PEDOT:PSS with respect to the slime mould cell, confirming evidences reported in literature for this polymer when interfaced with several kinds of bio-systems, *e.g.* proteins,^38^ cells,^19^ and bacteria.^39^

Fig. 2D–F report the typical PPC-OECT transfer characteristics (*I*_ds_ *vs. V*_gs_, *V*_ds_ = –0.4 V) comparing once again the performance of Ag, Pt and Au gate electrodes. The hysteresis loops were investigated by recording the transfer characteristics in a cyclic mode, *i.e.* using a scan sweep with increasing gate voltage followed by a backward sweep. In particular, *V*_gs_ was varied between –2 to 2 V, starting from 0 V, with step voltages of 0.2 V, at different bias step durations, *e.g.* 2, 5 and 10 s. In the positive range (0 < *V*_gs_ < 2 V) the action of *V*_gs_ induces an incorporation of cations into the PEDOT:PSS, causing its de-doping. Hence we observe a decrease of the channel current up to a saturation of the curves that depends on the gate material. In the negative range of the gate bias (–2 < *V*_gs_ < 0 V) we observe in all cases that a higher gate voltage is needed to establish the original doping level of the PEDOT:PSS channel, *i.e.* for the cations to desorb from the polymer backbone towards the PPC. In particular, in the case of an Ag-gate, the change of the backward current is quite small in the negative *V*_gs_ range, while it saturates at –1.5 V for the Pt-gate electrode and at about –2 V for the Au-gate electrode. Such behaviour suggests that chemical processes occur at the gate/PPC interface. The *I*_ds_ current flowing in the polymeric channel at *V*_gs_ = –2 V corresponds to the polymer *intrinsic current*. This current value is hundreds of μA higher than at zero-gate voltage, therefore, in the negative branch of the hysteresis curves, the ionic diffusion from the PEDOT:PSS towards the PPC is assisted by *V*_gs_. The high negative *V*_gs_ required to (re-)dope the polymer could be understood by taking into account the fact that the mould cell can be thought of as a highly viscous electrolyte, and that ions should pass through the cell membrane to control/modify the polymer state. The overall behaviour resembles that of a gel phase, and this would indicate that PPC works as a “quasi” solid electrolyte.^40,41^ This is an oversimplified picture since it does not consider that PPC is a cell and, consequently, for the actual mechanisms involved in the release/incorporation of ions, one should take into account more complex membrane mechanisms. Such biological mechanisms require further investigation and are currently an object of study.

A complementary observation concerns the area of the hysteresis curves, which have been found to be dependent on the gate material. In particular, the lowest area was found for the Ag-gate and the largest one for the Pt-gate electrode. In general, hysteresis arises from the competition between the dynamics of cation adsorption/desorption and the timescale with which the doping/de-doping occurs.^10,42^ The increase of the hysteresis area is hence related to the different operating regimes under which the OECT works according to the material of the gate electrode. More specifically, as already mentioned, the Ag-OECT operating regime (with a saline electrolyte) is almost fully Faradaic, and is characterized by a negligible potential drop at the gate/electrolyte interface,^35,36^ while with Au and Pt gates, OECTs are expected to work mostly in a capacitive operation mode (non-Faradaic mode), above all at lower gate voltages. It is worth noting that for Pt and Au (inert electrode materials) the channel current *I*_ds_ (in the range of mA) is much higher than the gate current *I*_gs_ (in the range of μA). Instead, when Ag is the gate electrode, *I*_gs_ is considerably higher (on average, by a factor of 5), this being the fingerprint of a Faradaic regime.^17^

A similar consideration holds for the *I*_ds_ steady-state level (saturation) in the positive range of *V*_gs_. Fig. 2–F shows that the Ag-gate gives a full-saturation level already at *V*_gs_ < +1 V, a value that is achieved at higher voltages with Au (about 1.8 V), while a real onset of saturation is not observed for Pt in the range studied. This behaviour further indicates possible electrochemical reactions taking place at the PPC/Ag-gate electrode (as already indicated by the curves in Fig. 1B). In fact, we expect that a Faradaic reaction at the Ag-gate electrode results in a higher modulation and a fast response of the device, that is in faster channel current decrease (de-doping phase) and enhancement (desorption of cations from the channel). These are exactly the trends observed in the data of Fig. 1B.

The main question here is on how the PPC affects the channel current of the device, that is the conductive state of the PEDOT:PSS thin film. Specifically, the de-doping mechanism induced by the injection of cations from the electrolyte upon gate biasing should be completely reversible, so that the subsequent doping process (as observed in Fig. 2D–F) can actually take place after a de-doping process. Since the hole density in the PEDOT:PSS film is influenced by the dopant density, that is the ionic concentration of the electrolyte,^43^ the transfer characteristics of Fig. 2D–F indicate that the PPC acts as a reservoir of cations which can be exchanged with the PEDOT:PSS film upon suitable gate biasing. We envisage that since the cell membrane (5 nm thick) is in contact with the PEDOT:PSS film, the cations contained in the intracellular matrix can cross the cell membrane, possibly through the ion channels under proper polarization, both towards and from the PEDOT:PSS, and this cationic motion allows to de-dope/dope the polymer, respectively. Cations that cross the cell membrane towards the PEDOT:PSS are driven by the applied *V*_gs_ > 0 V, so their motion will be faster than that of cations diffusing back from PEDOT:PSS to the cell membrane when *V*_gs_ = 0 V.

This is a good indication that the PPC-OECT response could be related to the transmembrane mobility of ions contained in the cell. In order to assess the specificity of the response induced by the cell, and hence the ability of our system to study such effects, we made comparative measurements with standard physiological solutions.

Fig. 2G–I show the results related to the transfer characteristics measured with 0.15 M NaCl as the electrolyte, and using the same procedures adopted for the corresponding curves recorded in the case of the PPC-OECT (Fig. 2D–F). Of particular interest are the differences observed in the transfer characteristics, where the hysteresis loops are clearly different from those obtained with PPC and reported in Fig. 2D–F. In particular, the following differences can be observed in the hysteresis loops:

(i) Shape: the transfer curves for both NaCl and PPC-based devices clearly show a specific shape dependence on the gate material; in particular, in the case of PPC-OECT, a widening of the hysteresis loop is observed. The maximum widening is centred at different *V*_gs_ values, according to the gate material (–0.5, 0, –1.2 V for Au, Pt, and Ag, respectively); this effect could be ascribable to the specific reaction of the intracellular matrix of the PPC with the gate material, resulting in a fingerprint of the electrochemical state of the PPC interior.

(ii) The transfer curves measured with NaCl show a progressive current decay upon measurement cycles, due to an over-oxidation effect induced by the largest bias reached in each cycle (+2 V);^44^ on the other hand, the PPC-OECT shows highly reproducible transfer curves with no loss of conductivity of PEDOT:PSS in the bias range investigated, resulting in a peculiar feature of the metallic gate/PPC/PEDOT:PSS system.

(iii) There are changes in the oxidation potential which are quite relevant, in particular for the Pt-gate electrode.

On the basis of these observations, we have significant indication that the OECTs based on a living cell/PEDOT:PSS interface sensitively monitor intracellular processes. This aspect will be a subject of future studies.

**Fig. 3:**
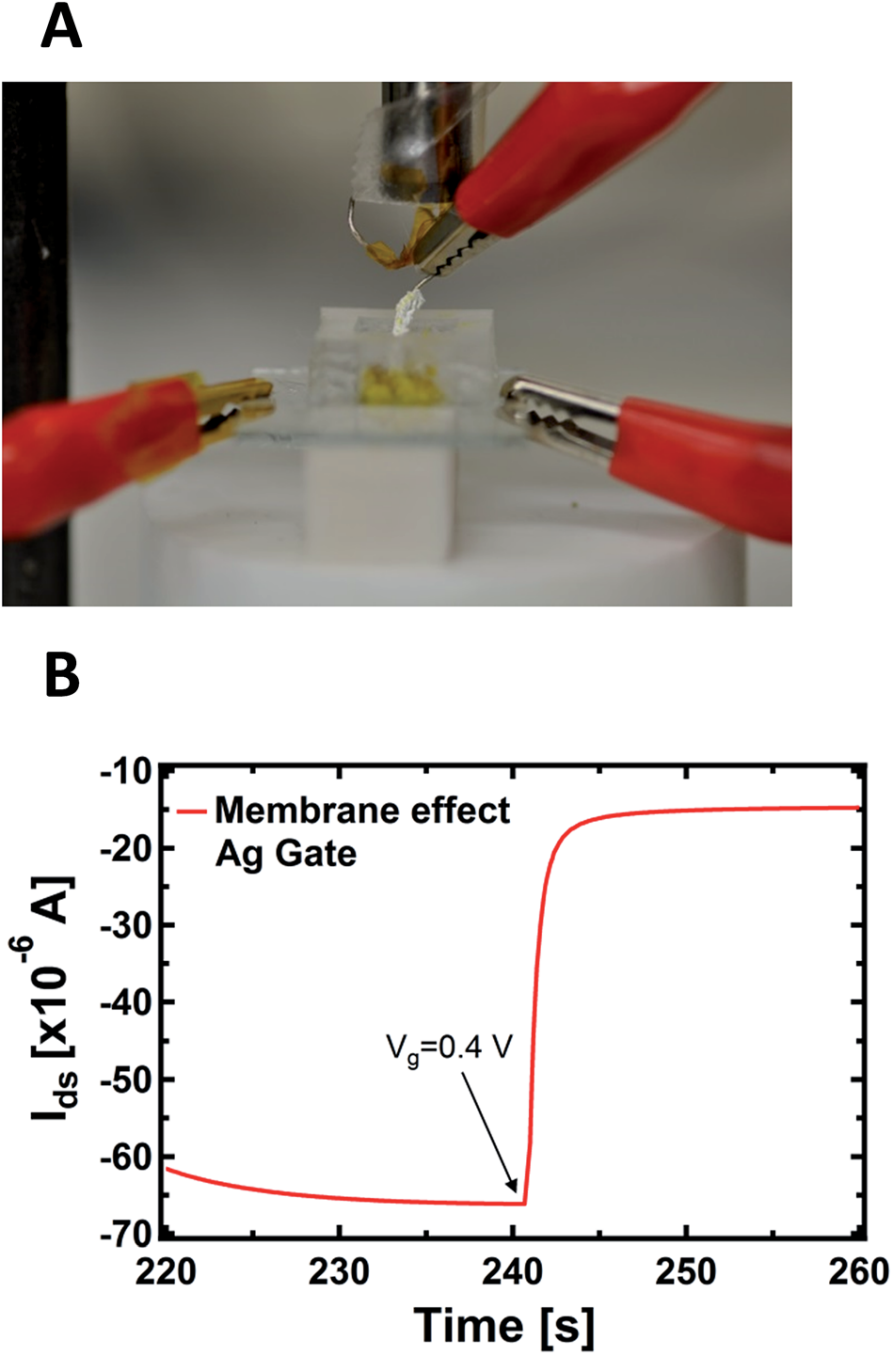
(A) Photo of the PPC-OECT device where the slime mould cell (yellow) and the gate electrode body covered by a Teflon film are visible. (B) Kinetic curve acquired with the protected gate; at the time of 240 s the gate voltage (*V*_gs_ = +0.4 V) was turned on.

An important aspect regards the fact that the PPC membrane in contact with the PEDOT:PSS is a wet surface composed of a complex environment, formed by ions, but also by bacteria and other species (different substances present in the body of the slime mould). It is not known *a priori* whether the doping/de-doping of PEDOT:PSS is mainly caused by cations coming from the wet surface of the membrane instead of the inside of the cell. To corroborate the idea that cations involved in the doping/de-doping of PEDOT:PSS come from the inside of the cell, we isolated the Ag gate-wire body with a Teflon film, except its apex. Then, we recorded the kinetic curves again in order to rule out the possible role of the membrane in terms of the device operation, since in this way the exposed tip of the metallic gate is in contact exclusively with the inside of the cell and the membrane does not experience the applied bias (Fig. 3A).

As reported in Fig. 3B, a single step of the resulting kinetic curve, recorded by applying *V*_gs_ = +0.4V at *t* = 240 s, shows that the current modulation is mainly due to the ionic intracellular content of the cell and does not depend on its wet external environment, which is in direct contact with the device channel. This is because the rising time of both the unexposed (Fig. 1B) and isolated electrodes (Fig. 3B) are comparable and no ionic transmembrane delay has been found in the latter case. This is in agreement with previous results dealing with the effect in the time domain of artificial lipid membranes on the device response.^45,46^ Moreover, this corroborates the idea of using such a device configuration to directly study the cell membrane response to pathologies and/or external agents,^47^ such as pore forming toxins.^48^ It is important to note that the repeated application of voltages to the PPC, in the range explored here with the gate immersed into the membrane, does not induce any stress or affect the viability of the cell.

### The OECT-PPC memristive device

**Fig. 4:**
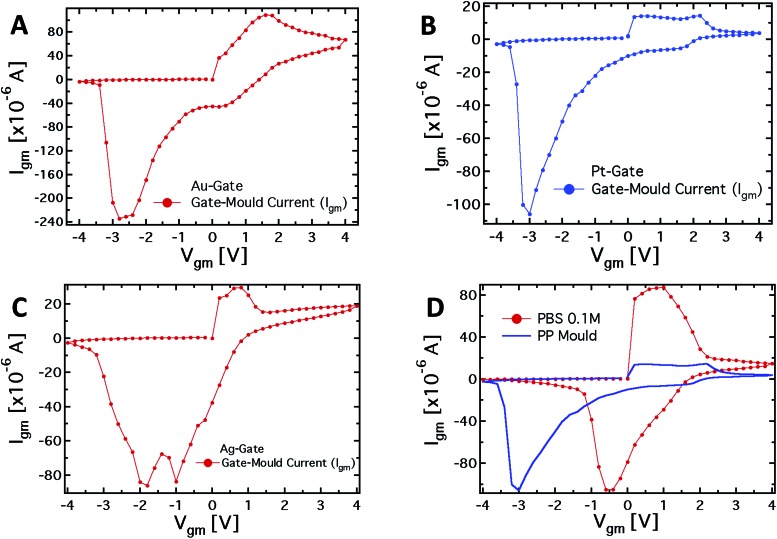
(A–C) Plots of the *I*–*V* measurements for 3 different working electrodes (Pt, Au and Ag) and with the PEDOT:PSS stripe used as the reference electrode. The voltage applied spans from –4 to +4 V, with a 0.2 V step. (D) Plot of the *I*–*V* measurement with a Pt-gate electrode with the mould and with a standard PBS 0.1 M electrolyte.

To further investigate the role of the gate electrode/PPC interface and to demonstrate the memristive properties of the device, we performed a conventional electrochemical study. To this aim, the same device was used in a 2-terminal configuration, with the PEDOT:PSS stripe as the reference electrode and a bias voltage applied between the gate, acting as working electrode, and one of the channel electrodes. Fig. 4A–C show the *I*–*V* curves for Au, Pt and Ag working electrodes, respectively.

The *I*–*V* curve in the case of the Pt-electrode shows a reduction peak at about –3 V and a broad oxidation peak, between +0.1 and +2.1 V. The reduction peaks observed for Au and Ag electrodes are located at about –2.8 and –1.9 V, respectively, while narrower oxidation peaks are located at +1.6 V and +0.7 V, respectively. The trend of the redox peaks is consistent and explains the behaviour shown in Fig. 2D–F. In fact, the saturation observed in the transfer curves in the positive gate voltage branch corresponds to the oxidation of the related gate electrode (+0.6 V for Ag gate, Fig. 2F), so that in the analysed gate voltage range, a Faradaic reaction is occurring at the Ag-gate electrode. Similarly, the onset of the channel current saturation in the transfer curve for the Au electrode is at about +1.6 V (Fig. 2D), while for Pt no onset of saturation is observed in the voltage range studied, confirming that oxidation of the electrode is not complete below +2.2 V (Fig. 2E).

It is worth noting that the oxidation peak for the Pt-electrode (Fig. 4B) seems to be the convolution of those obtained using Ag and Au electrodes. Pt electrodes, although substantially inert, are in fact also able to sustain Faradaic reactions in the presence of biomolecules,^49^ and the cytoplasm of the PCC is surely an environment rich in complex molecular species.

As far as the electrode reduction is concerned, the strong separation between reduction peaks with respect to the overlapped oxidation peaks and their own separation lead to a memristive-like behavior for our device. The memristive behavior can be attributed to a competition between the capacitive coupling at the PPC/PEDOT:PSS interface and the choice of the working electrode that promotes a flux of ionic species towards the underlying polymer. This flux is sustained to a greater or a smaller extent, depending on whether or not a Faradaic reaction takes place at the working electrode. In detail, since PPC is a macroscopic multinucleate single cell, two opposite features of the cell membrane characterize this system. First, in their simplest form cell membranes are phospholipid bilayers, showing a selective permeability with respect to ions and neutral molecules through the formation of ionic channels; in particular, leakage channels and voltage-sensitive channels that can be opened and closed in response to the applied voltage across the membrane, as happens for example in the case of the electroporation technique.^50^ Secondly, the membrane promotes the formation of an electric double layer at its edges,^51^ thus a capacitive coupling between the PPC and the PEDOT:PSS thin film is expected to be promoted. The strength of the external driving force (*i.e.* the applied voltage), responsible for the ionic flux through the cell membrane, is strictly connected to the redox reactivity of the electrode.^17,49^ On the other hand, the restoration of the initial state (*i.e.* the electrode reduction) in its early stage, that is when the external voltage decreases but is still positive, is assisted by the concentration gradient generated during the electrode oxidation between the inner and outer part of the cell. To better corroborate this idea, *I*–*V* curves have been acquired using a conventional electrolyte (*i.e.* a buffer solution, PBS) in place of PPC (reported in Fig. 4D). These curves, if compared with those obtained using the PPC cell, do not show a convincing memristive response: there is no evidence of stable switching between two well-defined and stable conductive states, as the oxidation and reduction peaks strongly overlap. In fact, when PEDOT:PSS/liquid electrolyte interfaces are promoted, during the restoration of the initial electrochemical state (reduction reaction) ions are evidently free to repopulate the liquid electrolyte. In this case, within a simplified picture, the concentration gradient effectively assists the electrolyte repopulation, favoring the reversibility of the electrochemical process and inducing, consequently, the overlapping between oxidation and reduction peaks. In addition, the residual negative current showed by *I*–*V* curves at *V* = 0 during the electrode reduction reaction, is a fingerprint of the electrolyte repopulation by the ions injected into the polymer during the electrode oxidation. This residual current in the case of the PPC is lower than that of the saline buffer PBS and shows a dependence on the chosen electrode. A salient aspect emerging from the above analysis concerns the classification of our memristor in terms of the relevant features characterizing an ideal memristor. In this respect, as stated by Chua,^4,52^ some fingerprints should be exhibited by an ideal memristor. The first fingerprint is constituted by the pinching of the *I*–*V* curve at the axis origin. In our case, this fingerprint is not completely fulfilled, but it is clear that the choice of the electrode is crucial for controlling this memristive feature. Therefore, the choice of an appropriate working electrode can induce a larger separation among the reduction peaks and, consequently, a more ideal behavior in terms of the first Chua's fingerprint. As the second fingerprint according to Chua's classification, a memristive device should exhibit a dependence of the hysteresis lobe area on the frequency of the applied periodic external signal, and the device output should tend to a single-valued function through the origin, when the frequency of this signal tends to infinity. In our case, the applied staircase voltage with different durations of the scan sweep represents a triangular waveform voltage sweep with varying frequency. A preliminary study (not shown) has indicated that the area of hysteresis loops decreases by a factor 2 if the frequency of the biasing signal is increased from 2.5 × 10^–3^ Hz to 1.25 × 10^–2^ Hz. Currently, we are systematically studying the features of our memristive device and the strategies aimed at optimizing its response.

From the observed response, our system can be classified quite well within the generalized Chua model, even though it cannot fully be considered as an ideal memristor. Actually, it is worth noting that none of the memristive systems reported in literature can be considered as an “ideal memristor”, since several processes are always responsible for their properties.^53^ However, the properties shown by the characteristics reported in Fig. 4A–C, that is the presence of the hysteresis loop and the rectification, allow us to consider the system under analysis as a memristive device in a wide sense. Regarding the device performance, the current values (*I*_gm_) for positive and negative biases measured in correspondence to the redox peaks, upon biasing lasting 10 seconds, allow for calculation of a rectification parameter (defined as *I*Oxgm/*I*Redgm) of 2.9, 2.2 and 7.4 for the Ag, Au and Pt-based structures, respectively.^49^ Of course, the difference in the working principles determines the difference in the observed characteristics, comparing them with those of titanium oxide systems, polyaniline-based devices, and even memristive devices based on a pure PPC.^54^ However, at least with respect to the pure *Physarum*-based device, the memory effect in our case is much more pronounced due to the modulation of the organic semiconductor layer conductivity. Making a comparison with the polyaniline-based devices, the suggested system has an advantage in the switching velocity: 1 s in the present case, compared with about a minute in the case of polyaniline memristive devices. Finally, comparing with oxide memristors, the organic nature of our system allows better biocompatibility and, therefore, easier integration into living organisms. In addition, the advantage of using a PPC-OECT compared with previously reported memristor devices is that the transition to the conductive state takes place as soon as a *V*_gm_ > 0 V is applied. At present PPC-OECT works efficiently and reliably as a memristor element for more than ten cycles before the natural degradation of the organic polymer starts depleting the device performance.

Finally, a very important point we would like to make here is that the PPC-OECT is a good candidate for an innovative memristive element since it satisfies the requirements sought for memristors, defined as electronic elements with memory properties. Recent literature considers the memristor as a viable circuital element for the manufacturing of bio-inspired information processing systems, of adaptive bio-inspired electronic networks (neuromorphic systems) and for mimicking learning capability.^55^

## Conclusions

In conclusion, we have demonstrated a fully working hybrid bio-organic OECT device, where the electrolyte is efficiently replaced by a living cell, the *Physarum polycephalum* cell (PPC), that can be operated as a memristor device. The semiconducting polymer PEDOT:PSS is used both as the transistor channel and as the reference electrode of a memristive device. The PPC-OECT is stable and reliable and has been characterized with 3 different electrodes (Pt, Au and Ag) used as the gate under the transistor-mode of operation and for monitoring the cell activity. The PPC-OECT device response is quite sensitive to the presence of the PPC on the surface of PEDOT:PSS and envisages the potential that the response curves in transistor mode could yield information about the PPC state and properties. Moreover, *I*–*V* measurements give insight into the memory capabilities of the mould, showing a memristive response ascribable to the cell membrane properties.

In a broader perspective, we believe that the device proposed here can be classified as a Bio-Organic Sensing/Memristive Device (BOSMD) that enables new and unexplored opportunities in bioelectronics. In particular, for the first time the unique properties of cellular systems are interfaced with the electronic/ionic responses that characterize OECTs. Such a combination is uniquely suitable to explore and efficiently produce bioelectronic actions combining the bifunctional transistor/memristor response and the sensing properties. In fact, the integration of PPC with OECTs could in principle allow the direct monitoring of the internal cellular bioactivities, including cell metabolism and the reactions/interactions with environmental changes. The electrical transduction of such processes and the control (both ionically and electronically) of the mentioned “smart” functions that PPC is capable of (if possible, extended to other cells or bioactive systems too), pave the way to devices enabling remarkable novel activities such as responding to external stimuli and changing their structural/chemical properties, as living beings do (examples in the case of PPC are the development of a protoplasmatic network assembly in a well-ordered manner that can be exploited as an electrical array, or the ability of PPC to change its 3-dimensional shape in order to catch food). Furthermore, the BOSMD being a device simultaneously sensitive and capable of memorizing previous activities, it offers jointly the unprecedented ability to mimic the behaviour of living organisms, to study their interaction with the environment and, ultimately, to implement new neuromorphic systems. Thus, we consider the PPC-OECT proposed here as a prototype of BOSMDs that are a family of hybrid living–artificial man made devices.^40^ The present work is a starting point for the development of such kinds of devices. We have demonstrated here only the feasibility of PEDOT:PSS transistors and memristive devices with living beings. Further efforts will be directed to the particular realization of multi-sensitive elements, providing an integral response to *Physarum* metabolism as a result of variations in the environmental conditions. In parallel, we plan to explore the memory effects, when the growth of the PPC will vary the properties of individual devices and their mutual connections in related networks.

## References

[cit1] Adamatzky A. and Komosinski M., Artificial Models in Hardware, 2009.

[cit2] Mann S. (2008). Angew. Chem., Int. Ed..

[cit3] Erokhin V., Fontana M. P. (2011). J. Comput. Theor. Nanosci..

[cit4] Chua L. (1971). IEEE Trans. Circuit Theory.

[cit5] Strukov D. B., Snider G. S., Stewart D. R., Williams R. S. (2008). Nature.

[cit6] Malliaras G. G. (2013). Biochim. Biophys. Acta.

[cit7] Richter-Dahlfors A., Svennersten K., Larsson K. C., Berggren M. (2011). Biochim. Biophys. Acta, Gen. Subj..

[cit8] Tarabella G., Mahvash Mohammadi F., Coppede N., Barbero F., Iannotta S., Santato C., Cicoira F. (2013). Chem. Sci..

[cit9] Organic Electronics: Emerging Concepts and Technologies, ed. F. Cicoira and C. Santato, WILEY-VCH Verlag GmbH, Weinheim, Ge, 2013.

[cit10] Tarabella G., Balducci A. G., Coppedè N., Marasso S., D'Angelo P., Barbieri S., Cocuzza M., Colombo P., Sonvico F., Mosca R., Iannotta S. (2013). Biochim. Biophys. Acta, Gen. Subj..

[cit11] Jimison L. H., Tria S. A., Khodagholy D., Gurfinkel M., Lanzarini E., Hama A., Malliaras G. G., Owens R. M. (2012). Adv. Mater..

[cit12] Yao C., Xie C., Lin P., Yan F., Huang P., Hsing I.-M. (2013). Adv. Mater..

[cit13] Khodagholy D., Doublet T., Quilichini P., Gurfinkel M., Leleux P., Ghestem A., Ismailova E., Hervé T., Sanaur S., Bernard C., Malliaras G. G. (2013). Nat. Commun..

[cit14] Lanzani G. (2014). Nat. Mater..

[cit15] Tybrandt K., Gabrielsson E. O., Berggren M. (2011). J. Am. Chem. Soc..

[cit16] Tybrandt K., Forchheimer R., Berggren M. (2012). Nat. Commun..

[cit17] Tarabella G., Santato C., Yang S. Y., Iannotta S., Malliaras G. G., Cicoira F. (2010). Appl. Phys. Lett..

[cit18] Nilsson D., Chen M., Kugler T., Remonen T., Armgarth M., Berggren M. (2002). Adv. Mater..

[cit19] Lin P., Yan F., Yu J., Chan H. L. W., Yang M. (2010). Adv. Mater..

[cit20] Khodagholy D., Curto V. F., Fraser K. J., Gurfinkel M., Byrne R., Diamond D., Malliaras G. G., Benito-Lopez F., Owens R. M. (2012). J. Mater. Chem..

[cit21] Tarabella G., Nanda G., Villani M., Coppede N., Mosca R., Malliaras G. G., Santato C., Iannotta S., Cicoira F. (2012). Chem. Sci..

[cit22] Khodagholy D., Doublet T., Gurfinkel M., Quilichini P., Ismailova E., Leleux P., Herve T., Sanaur S., Bernard C., Malliaras G. G. (2011). Adv. Mater..

[cit23] Baumgarten W., Hauser M. J. B. (2010). Interdiscip. Sci.: Comput. Life Sci..

[cit24] Dussutour A., Latty T., Beekman M., Simpson S. J. (2010). Proc. Natl. Acad. Sci. U. S. A..

[cit25] Whiting J. G. H., de Lacy Costello B. P. J., Adamatzky A. (2014). Sens. Actuators, B.

[cit26] Tsuda S., Aono M., Gunji Y.-P. (2004). Biosystems.

[cit27] Adamatzky A., Jones J. (2011). Biophys. Rev. Lett..

[cit28] Schumann A., Adamatzky A. (2011). New Math. Nat. Comput..

[cit29] Berggren M., Richter-Dahlfors A. (2007). Adv. Mater..

[cit30] Isaksson J., Kjall P., Nilsson D., Robinson N. D., Berggren M., Richter-Dahlfors A. (2007). Nat. Mater..

[cit31] Crispin X., Marciniak S., Osikowicz W., Zotti G., van der Gon A. W. D., Louwet F., Fahlman M., Groenendaal L., De Schryver F., Salaneck W. R. (2003). J. Polym. Sci., Part B: Polym. Phys..

[cit32] Ouyang J., Xu Q., Chu C.-W., Yang Y., Li G., Shinar J. (2004). Polymer.

[cit33] Cifarelli A., Dimonte A., Berzina T., Erokhin V. (2014). Bionanoscience.

[cit34] Zhu Z. T., Mabeck J. T., Zhu C., Cady N. C., Batt C. A., Malliaras G. G. (2004). Chem. Commun..

[cit35] Bernards D. A., Macaya D. J., Nikolou M., DeFranco J. A., Takamatsu S., Malliaras G. G. (2008). J. Mater. Chem..

[cit36] Cicoira F., Sessolo M., Yaghmazadeh O., DeFranco J. A., Yang S. Y., Malliaras G. G. (2010). Adv. Mater..

[cit37] Lin F., Lonergan M. C. (2006). Appl. Phys. Lett..

[cit38] Wan A. M. D., Schur R. M., Ober C. K., Fischbach C., Gourdon D., Malliaras G. G. (2012). Adv. Mater..

[cit39] He R.-X., Zhang M., Tan F., Leung P. H. M., Zhao X.-Z., Chan H. L. W., Yang M., Yan F. (2012). J. Mater. Chem..

[cit40] Adamatzky A., Physarum machine: Computers from slime mould, World Scientific Publishing, 2010, vol. 74.

[cit41] Kamiya N., Kuroda K. (1958). Protoplasma.

[cit42] Larsson O., Laiho A., Schmickler W., Berggren M., Crispin X. (2011). Adv. Mater..

[cit43] Bernards D. a., Malliaras G. G. (2007). Adv. Funct. Mater..

[cit44] Romeo A., Dimonte A., Tarabella G., D'Angelo P., Erokhin V., Iannotta S. (2015). APL Mater..

[cit45] Bernards D. A., Malliaras G. G., Toombes G. E. S., Gruner S. M. (2006). Appl. Phys. Lett..

[cit46] Berzina T. S., Troitsky V. I., Vakula S., Riccio A., De Rosa M., Nicolini C. (1997). Mater. Sci. Eng., C.

[cit47] Szachowicz-Petelska B., Dobrzyńska I., Figaszewski Z. A. (2014). Adv. Biol. Chem..

[cit48] Zanetti M., Maniglio D., Fasoli C., Pola M., Borga E., Corradi C., Dalla Serra M., Iannotta S., Motta A., Toccoli T. (2014). Electroanalysis.

[cit49] Tarabella G., Pezzella A., Romeo A., D'Angelo P., Coppede N., Calicchio M., d'Ischia M., Mosca R., Iannotta S. (2013). J. Mater. Chem. B.

[cit50] Neumann E., Schaefer-Ridder M., Wang Y., Hofschneider P. H. (1982). EMBO J..

[cit51] Uehara M., Sakane K. K., Maciel H. S., Urruchi W. I. (2000). Am. J. Phys..

[cit52] Adhikari S. P., Sah M. P., Kim H., Chua L. O. (2013). IEEE Trans. Circuits Syst..

[cit53] Pershin Y. V., Di Ventra M. (2011). Adv. Phys..

[cit54] Gale E., Adamatzky A., Costello B. D. L. (2014). arXiv.

[cit55] Erokhin V., Berzina T., Smerieri A., Camorani P., Erokhina S., Fontana M. P. (2010). Nano Commun. Netw..

